# The protective role of life satisfaction, coping strategies and defense mechanisms on perceived stress due to COVID-19 emergency: A chained mediation model

**DOI:** 10.1371/journal.pone.0242402

**Published:** 2020-11-13

**Authors:** Alessio Gori, Eleonora Topino, Annamaria Di Fabio

**Affiliations:** 1 Department of Health Sciences, University of Florence, Florence, Italy; 2 Department of Human Sciences, LUMSA University of Rome, Rome, Italy; 3 Department of Education, Languages, Interculture, Letters and Psychology (Psychology Section), University of Florence, Florence, Italy; Universita degli Studi di Bari Aldo Moro, ITALY

## Abstract

The coronavirus disease-19 (COVID-19) pandemic represents a worldwide emergency, which may have harmful consequences on people’s mental health. Parallel to research focused on risk factors, it could be useful to investigate the factors that help to cope with such crises at an emotional level. Therefore, this study aimed to strengthen the role of variables that protect from subjective distress during the COVID-19 pandemic, explore the pathways between satisfaction with life and perceived stress, and consider the role of coping strategies and defense mechanisms in this relationship. A sample of 1102 Italian participants who were experiencing the COVID-19 lockdown measures (Mage = 34.91, SD = 11.91) completed an online survey in which the Ten Item Perceived Stress Scale, Satisfaction with Life Scale, Coping Orientation to Problems Experienced Inventory and Forty-Item Defense Style Questionnaire were included. The data were analyzed using Pearson’s r correlations and moderation analysis. A chained-mediation model showed that the relationship between life satisfaction and perceived stress is partially mediated by approach coping, positive attitude and mature defenses. This study contributes toward gaining a better understanding of a protective pathway for mental health outcomes during the COVID-19 pandemic. The findings could be useful from both a preventive and an intervention perspective.

## Introduction

The coronavirus disease-19 (COVID-19) is a highly contagious disease caused by the spread of the Coronavirus 2 (SARS-CoV-2), and represents a serious global public health emergency [1]. It may produce a severe illness that, in the most critical cases, may progress and lead to respiratory failure and multi-organ issues [2], sometimes lethal [3]. Given the physical health risks, drastic measures across all continents were implemented leading to national lockdowns and border closures. However, parallel to the strictly medical issues, many studies drew attention to the consequences on mental health, since people emotionally affected by the pandemic exceeded those who have been diagnosed with COVID-19 [4]. Indeed, research on previous epidemics, such as the SARS (2002–2004) and Ebola (2013–2016), proved the association between these outbreaks and significant levels of psychological distress [5, 6]. This is consistent with recent studies highlighting the increase in perceived stress and mental health diseases related to the COVID-19 pandemic [7–10] and the resulting preventive measures (see [11] for a review): the risk of infection, financial difficulties, lockdown and physical distancing measures, were associated with moderate and severe stress [12] and emotional distress [13], linked to psychological and behavioral symptoms such as anxiety, fear, somatic disorders, sleep problems and increased use of substances, especially tobacco and alcohol [14–16]. Furthermore, this could have a negative impact on health that could persist even after the emergency is over, in light of the significant contribution of perceived stress to short and long-term psychophysical impairments (see [17] for a review), such as cardiovascular diseases [18], weakened immune responses [19], chronic pain [20], post-traumatic stress, and depression [21]. Given the range of the pandemic events, many studies on COVID-19 have focused on negative outcomes. However, scientific literature [22] also highlights the presence of variability in the responses to potentially stressful events. This opens a way to the study of factors that could play a protective role in psychological health during and after the pandemic.

With this in mind, a previous study showed that satisfaction with life was related to better mental health outcomes related to COVID-19 [23]. Indeed, it could be seen not only as a result deriving from different dispositional and contextual aspects (see [24] for a review) but also as a factor, in turn, responsible for many life outcomes [25–27]. It is a core component of subjective well-being [28] associated with lower perceived stress [29] and to lesser effects thereof [30], as well as to more positive behaviors in favor of one’s health [31] and, consequently, better physical health [32].

Based on the above-described scenario, the present study aimed to deepen the link between satisfaction with life and perceived stress in individuals who are experiencing the lockdown due to the global COVID-19 pandemic, also focusing on the role of coping and defense mechanisms in catalyzing this protective path.

Coping styles and defense mechanisms were classified as two independent key resources for stress-adaptation processes [33]. Coping strategies could be defined as “basic categories used to classify how people react to or handle stress” [34] (p. 875). Scientific literature has suggested that effective coping styles could promote emotional wellbeing, self-esteem, and quality of life [35], while the ineffective ones were associated with higher distress, anxiety, and depression [36]. Previous studies showed a significant association between life satisfaction and problem-focused coping [37], and the active protective coping (such as washing hands frequently or wearing masks) was found to be a negative predictor of psychological diseases during the COVID-19 pandemic [16]. Moreover, problem-focused coping is related to positive attitude and, more specifically, its subdimension of positive reinterpretation [37]. It permits to consider difficult events from different more adaptive and positive perspectives, protecting from negative mental health outcomes [38, 39], as found in people who have experienced the COVID-19 quarantine [40]. Babore and colleagues [41] have consistently shown that a positive attitude was the greatest protective factor for perceived stress among healthcare professionals during the COVID-19 pandemic.

On the other hand, defense mechanisms have been defined as mental operations, generally unconscious and automatic, to protect the self from internal conflicts or stressful situations [42]. They are put in place when coping is exceeded and differ from each other both in terms of adaptation degree and psychological function [43]. In this regard, evidence has highlighted that immature and neurotic defenses are markedly and moderately associated with psychopathologic symptoms, respectively, while the mature ones have shown a strong and negative correlation [44].

Given this theoretical framework, the current study aimed to investigate the factors that may protect from perceived stress during the COVID-19 pandemic. Therefore, a chained mediation model was hypothesized, in which approach coping, positive attitude and mature defenses mediate the relationship between life satisfaction and perceived stress.

## Material and methods

### Participants and procedure

This study involved 1102 Italian participants (30% males and 70% females), with a mean age of 34.91 years (*SD* = 11.91; 18 to 88 years old). All individuals were recruited on the internet through a snowball-like procedure by spreading an anonymous link and they completed an online survey using the Google Form platform. The general aim of the study was explained and all participants provided informed consent electronically before starting. The respondents declared that they had not received a diagnosis of COVID-19 and they had not received any form of payment for participating. Privacy and anonymity were guaranteed. The survey was launched on March 15, 2020, and remained open until March 25 (a period corresponding to 10 days in the pandemic), during the period of national lockdown, in which the movement of the population was restricted, except for necessity, work, and health-related circumstances. The research protocol was approved by the Ethical Committee of the Integrated Psychodynamic Psychotherapy Institute (IPPI).

### Measures

#### Italian Satisfaction with Life Scale (I-SWLS)

The Satisfaction with Life Scale (SWLS) [45] is a self-report scale to assess global life satisfaction. It consists of five items (e.g., “*In most ways*, *my life is close to my ideal*.” or “*So far I have gotten the important things I want in my life*”), rated on a seven-point Likert scale, from 1 (Strongly disagree) to 7 (Strongly agree). The Italian version [46, 47] was used in this study and it showed a good internal consistency with a Cronbach’s α = .90 in the present sample, confirming the good psychometric properties of the scale.

#### Italian Ten Item Perceived Stress Scale (I-PSS-10)

The Ten Item Perceived Stress Scale (PSS-10) [48] is a ten-item self-report scale to assess the subjective perception of how stressful lived circumstances and events are. The instructions ask the respondents to indicate how often respondent has been felt as described by the items (e.g., “*Upset because of something that happened unexpectedly*” or “*Unable to control the important things in your life*”) in the last month on a five-point Likert scale, ranging from 0 (never) to 4 (very often). For the present study, the Italian translation of Fossati [49] was used, showing a good internal consistency (α = .88).

#### Coping Orientation to Problems Experienced—New Italian Version (COPE-NVI)

The Coping Orientations to Problems Experienced (COPE) [50] is a sixty-item self-report measure that assesses the respondents’ general coping strategies. In this study, the Italian version (COPE-NVI) [51] was used. All items are rated on a four-point Likert scale, from 1 (“I don't usually do this at all”) to 4 (“I usually do this”), and grouped into five dimensions, all with good Cronbach’s alpha in the present sample: 1) Social Support (12 items; α = .90), that is, the search for emotional or instrumental social support and the focus on and venting of emotions (e.g., “*I discuss my feelings with someone*”, “*I talk to someone to find out more about the situation*” or “*I get upset and let my emotions out*”); 2) Avoidance Strategies (16 items, α = .81), including denial, behavioral disengagement, mental disengagement and substance use (e.g., “*I refuse to believe that it has happened*”, “*I just give up trying to reach my goal*”, “*I turn to work or other substitute activities to take my mind off things*” or “*I use alcohol or drugs to make myself feel better*”); 3) Positive Attitude (12 items, α = .79), including positive reinterpretation, acceptance and restraint (e.g., “*I try to grow as a person as a result of the experience*”, “*I get used to the idea that it happened*” or “*I restrain myself from doing anything too quickly*”); 4) Approach Coping (12 items, α = .84), including suppression of competing activities, planning and active coping (e.g., “*I focus on dealing with this problem*, *and if necessary let other things slide a little*”, “*I make a plan of action*” or “*I concentrate my efforts on doing something about it*”); 5) Transcendent orientation (8 items, α = .83), indicating the tendency to humor or see religion as a source of support from an emotional perspective or a vehicle for growth (e.g., “*I laugh about the situation*” or “*I put my trust in God*”).

#### Italian Forty Item Defense Style Questionnaire (I-DSQ– 40)

The Forty Item Defense Style Questionnaire (DSQ– 40) [52] is a fourty-item self-report measure to assess defense mechanism on a nine- point Likert scale ranging from 1 (Strongly disagree) to 9 (Strongly agree). In this study, the Italian version of Farma and Cortinovis [53] was used, with three principal subdimension which showed acceptable internal consistency: 1) Mature defense style (8 items; e.g., “*I work out my anxiety through doing something constructive and creative like painting or woodwork*” or “*I'm usually able to see the funny side of an otherwise painful predicament*”; α = .55), consisting of sublimation, humor, anticipation, and suppression; 2) Neurotic defense style (8 items; e.g., “*If I have an aggressive thought*, *I feel the need to do something to compensate for it*” or “*I always feel that someone I know is like a guardian angel*”; α = .60), consisting of pseudo-altruism, idealization, and reaction formation; 3) Immature defense style (24 items; e.g., “*I get openly aggressive when I feel hurt*” or “*I fear nothing*”; α = .82), consisting of projection, acting out, isolation, devaluation, autistic fantasy, denial, passive aggressiveness, displacement, disassociation, splitting, rationalization, and somatization.

### Data analysis

The SPSS software (IBM-SPSS 25.0 version, IBM, Armonk, NY, USA) for Windows was used to analyze the data. Descriptive statistics for all the variables were calculated and Pearson’s r correlations were used to investigate the associations among them. Then, the hypothesized chained mediation model was tested, by using the macro-program PROCESS v. 3.4 [54]. Therefore, Model 6 was applied to explore direct and indirect paths in the relationship between Life Satisfaction and Perceived stress, including Approach Coping, Positive Attitude and Mature Defenses as mediators. Finally, the indirect effect was estimated by implementing the bootstrapping technique with 5000 bias-corrected bootstrap samples and the 95% confidence interval.

## Results

Table 1 shows descriptive statistics and Pearson’s correlation.

**Table 1:** Correlation matrix (N = 1102).

	1	2	3	4	5	6	7	8	9	10	*M*	*SD*
1) Satisfaction with life	1										21.88	6.87
2) Perceived stress	-.368^**^	1									19.03	7.69
3) Social support	.113^**^	.198^**^	1								30.73	7.95
4) Avoidance strategies	-.324^**^	.415^**^	.151^**^	1							25.97	6.42
5) Positive attitude	.280^**^	-.173^**^	.271^**^	-.020	1						30.71	5.37
6) Approach coping	.277^**^	-.133^**^	.344^**^	-.092^**^	.779^**^	1					30.66	6.33
7) Transcendent orientation	0.48	.125^**^	.094^**^	-.071^*^	-.046	-.044	1				19.10	5.10
8) Mature defenses	.176^**^	-.153^**^	.031	.085^**^	.410^**^	.353^**^	-.168^**^	1			43.43	9.08
9) Neurotic defenses	-.077^*^	.323^**^	.210^**^	.304^**^	.143^**^	.075^*^	.158^**^	.336^**^	1		33.89	9.85
10) Immature defenses	-.300^**^	.405^**^	-.032	.528^**^	-.005	-.017	-.012	.300^**^	.522^**^	1	95.00	26.09

**. Correlation is significant at the 0.01 level (2-tailed).

*. Correlation is significant at the 0.05 level (2-tailed).

Satisfaction with life correlated significantly and negatively with Perceived stress (r = -.368; p < .001). The latter showed other significant and negative association with Approach coping (r = -.133; p < .001), Positive attitude (r = -.173; p < .001) and Mature defenses (r = -.154; p < .001), which, in turn, were significantly and positively correlated with Satisfaction with life (r = -.133; p < .001, r = -.173; p < .001; r = -.154; p < .001, respectively). Approach coping and Positive attitude showed a high significant positive association (r = .779; p < .001) and they were significantly and positively related with Mature defenses (r = .353; p < .001; r = .410; p < .001, respectively).

**Fig 1:**
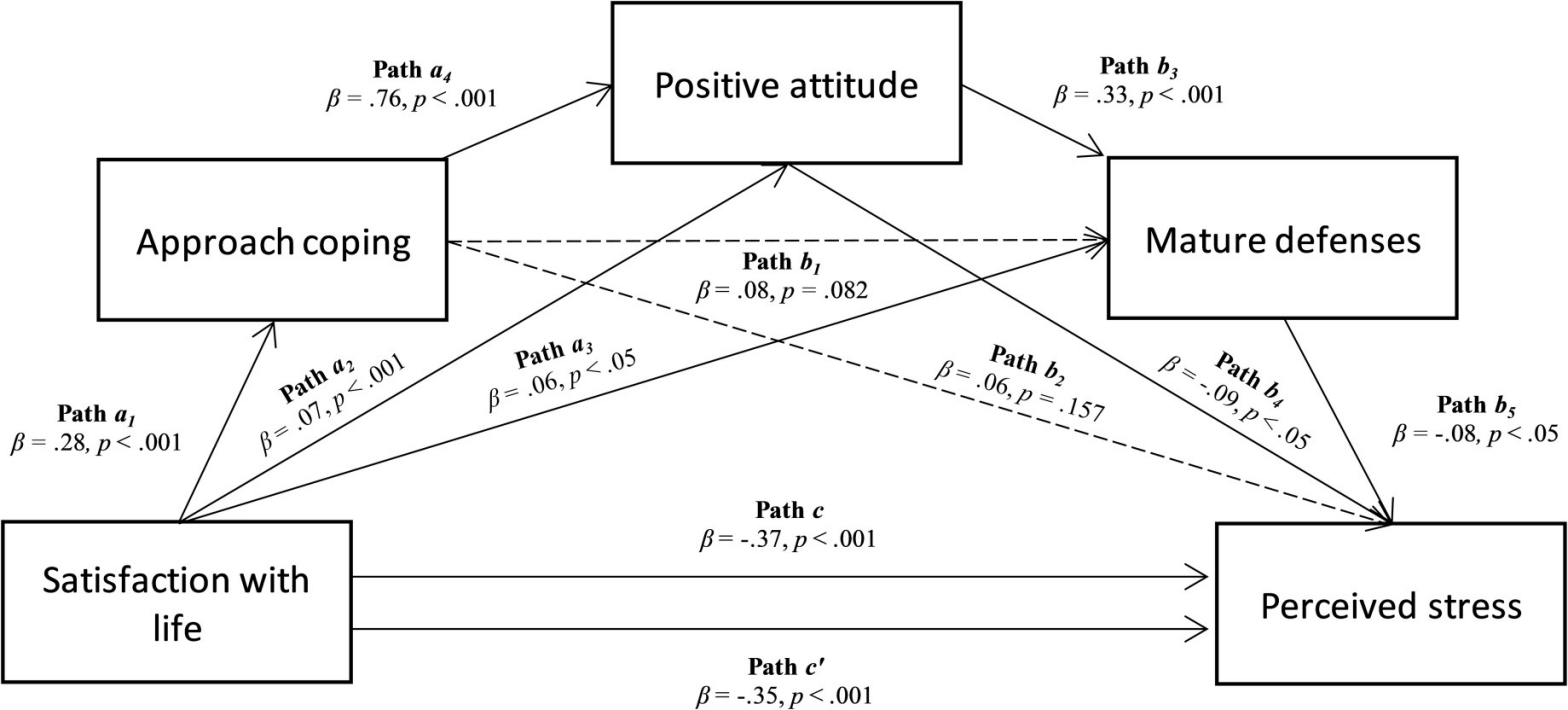
Chained mediation model. The mediation of approach coping, positive attitude, and mature defenses in the relationship between satisfaction with life and perceived stress.

The correlation outcomes supported the relationship between the variables included in the hypothesized model; therefore, that was tested (Fig 1).

The significant negative relationship between Satisfaction with life and Perceived stress was confirmed (path c; β = -.37, p < .001, Boot LLCI = -.473–Boot ULCI = -.350). Furthermore, Satisfaction with life was significantly and positively related to Approach coping (path a1; β = .28, p < .001, Boot LLCI = .203–Boot ULCI = .308), Positive attitude (path a2; β = .07, p < .001, Boot LLCI = .025–Boot ULCI = .085), and Mature defenses (path a3; β = .06, p < .05, Boot LLCI = .007–Boot ULCI = .156). Approach coping was significantly and positively associated with Positive attitude (path a4; β = .76, p < .001, Boot LLCI = .611–Boot ULCI = .677) which, in turn, was significantly and positively related with the use of Mature defenses (path b3; β = .33, p < .001, Boot LLCI = .418–Boot ULCI = .710). Both of these had a significant negative relationship with Perceived stress (path b4; β = -.09, p < .05, Boot LLCI = -.264–Boot ULCI = -.007 and path b5; β = -.08, p < .05, Boot LLCI = -.115–Boot ULCI = -.013, respectively). The effect of Satisfaction with life was reduced after controlling the mediators (path c’; β = -.37, p < .001, Boot LLCI = -.451–Boot ULCI = -.323), albeit remaining significant: this suggest a partial mediation, with R2 = 0.146, F(4, 1097) = 42.052, p < .001 (see Table 2).

**Table 2:** Mediation model coefficients (N = 1102).

	Consequent															
		M1				M2				M3				Y		
Antecedent		Coeff.	SE	*p*		Coeff.	SE	*p*		Coeff.	SE	*p*		Coeff.	SE	*p*
X	*a*^*1*^	.255	.027	< .001	*a*^*2*^	.055	.015	< .001	*a*^*3*^	.082	.038	.032	*c’*	-.387	.033	< .001
M1		-	-	-	*a*^*4*^	.644	.017	< .001	*b*^*1*^	.110	.063	.082	*b*^*2*^	.077	.054	.157
M2		-	-	-		-	-	-	*b*^*3*^	.564	.074	< .001	*b*^*4*^	-.136	.066	.039
M3		-	-	-		-	-	-		-	-	-	*b*^*5*^	-.064	.026	.014
Constant	*i*_*M1*_	25.069	.613	< .001	*i*_*M2*_	9.773	.536	< .001	*i*_*M2*_	20.972	1.506	< .001	*i*_*Y*_	32.072	1.409	< .001
		*R*^*2*^ = 0.077				*R*^*2*^ = 0.611				*R*^*2*^ = 0.175				*R*^*2*^ = 0.146		
		*F*(1, 1100) = 91.389, *p <* .001				*F*(2, 1099) = 826.307, *p <* .001				*F*(3, 1098) = 77.497, *p <* .001				*F*(4, 1097) = 42.052, *p <* .001		

***Note***: X = Satisfaction with life; M1 = Approach coping; M2 = Positive attitude; M3 = Mature defenses; Y = Perceived stress.

Finally, the significance of the indirect effect was probed by the bootstrapping procedure (Boot LLCI = -.012–Boot ULCI = -.001), confirming, the statistical significance of this chained mediation. All the indices concerning the model effects are shown in Table 3.

**Table 3:** Model effect indices (N = 1102).

Total Effect	Direct Effect	Indirect Effect	Partial Standardized Indirect Effect	Completely Standardized Indirect Effect	Bootstrapping 95% CI for Indirect Effect
-.412	-.387	-.006	-.001	-.005	[-.0121; -.0007]

## Discussion

The existing literature on the effects of the COVID-19 pandemic has highlighted the presence of significant psychological symptoms, with the risk of serious repercussions on global mental health in both the short and long terms (for a review see [55]). However, previous research supported the need to underline, together with the analysis of psychobiosocial outcomes linked to the spread of infectious diseases, also the factors that allow us to face such critical events at an emotional level [56]. Therefore, this study aimed to deepen the variables which may have a protective effect from subjective distress during the COVID-19 pandemic, exploring the pathways between satisfaction with life and perceived stress, also considering the role of approach coping, positive attitude, and mature defense mechanisms in this relationship.

As expected, based previous research [29], Pearson’s correlation matrix showed a negative relationship between perceived stress and satisfaction with life. With regard to coping strategies and defense mechanisms, only approach coping, positive attitude, and mature defenses were negatively related with perceived stress, while other constructs investigated were positively associated with it. These data represent further evidence of the different levels of coping and defense functionality in potentially traumatogenic life events, delineating a situation-based continuum of adaptivity with consequences for physical and emotional health [57]. Therefore, the results concerning the protective factors were explored in more depth in the chained mediation analysis. First, the protective role of satisfaction with life on perceived stress was confirmed. This is in line with previous research, suggesting that the perception of having a meaningful life, which is a key element for a sense of satisfaction [58], is associated with lower stress, healthy behaviors and more active and adaptive coping (for a review, see [59]). Data consistently showed an indirect pathway in which satisfaction with life was positively related to approach coping and positive attitude, as well as mature defense mechanisms, which were consecutively linked to each other. Finally, both a positive attitude and mature defenses played a significant role in containing perceived stress. Such findings could be read in light of the effect of positive attitude in attenuating stress reactivity [60], and in that of mature defense mechanisms, which facilitate acceptance and constructive management of uncomfortable feelings due to the COVID-19 pandemic [61] and mitigate distress [57].

Several limitations need to be highlighted to ensure the correct interpretation of the findings of this study. First, a snowball-like procedure of sampling was adopted. This was a “forced choice” due to lockdown impediments and limitations, which did not permit a random selection and an effective representation of the general population. Moreover, this study did not consider differences based on gender, culture, and socio-economic level. Future research could focus on these aspects, given the greater anxiety related to the virus found in women [10] or the privileges conferred by wealth in facing lockdown and social distancing in a comfortable place, with sufficient food and resources. The participants’ residential region, profession or possibility to have friends and relatives who had been infected, were not investigated. These could be important challenges for future research, in light of the different ways of the spread of the virus [62], the significant levels of stress found in healthcare workers (see [63] for a review) and the difficulties faced by the patients’ relatives [64]. Finally, the cross-sectional nature of the study implies the need to be cautious in interpreting the results. In fact, this does not allow us to understand whether the participants’ obtained scores reflected their baselines levels or were due to the experience of the COVID-19 crisis, although previous studies have shown that the pandemic is responsible for an increase in psychological symptoms (e.g. [7]). Moreover, no causal relationships could be clearly demonstrated. Therefore, longitudinal research will be needed to confirm and extend our results.

## Conclusions

This study contributes toward gaining a further important step toward the understanding of a protective pathway for mental health outcomes during the COVID-19 pandemic and provides useful support for furthering existing research in this area.

This worldwide state of emergency, in fact, can have significant repercussions on people’s emotional state and it is essential to expand the knowledge of the factors useful for designing interventions which may help to preserve psychological health during the pandemic [65]. These findings highlight the need for paying particular attention to satisfaction with life, but also approach coping, positive attitude and mature defenses, to contain perceived stress levels during the COVID-19 phases. From an intervention or prevention perspective, for example, life satisfaction may be implemented by focusing on both personal (e.g., improving character strengths) and contextual aspects (e.g., external support), with multicomponent programs (e.g., [66]). On the other hand, the results of the study also suggest the possibility and usefulness of the application of coping strategies’ focused training on the pandemic’s context, as promoted by Folkman e Greer [67], favoring the problem-focused coping and the meaning-based one. Finally, all this could be integrated with a focus on defense mechanisms, implementing their insight, mentalization and the use of more mature and adaptive ones [42]. These results may have practical implications in large-scale to preserve the mental health of individuals and people at risk and to improve the quality of psychological interventions. In this regard, for example, these results may offer guidelines for the support of healthcare professionals, who report significant levels of subjective distress [63], but also on people who ask for a psychological treatment because of the pandemic, such as individuals, families and workers [68]. Moreover, these findings provide a further piece in a growing theoretical panorama which supports the importance of life satisfaction, mature defense mechanisms and coping strategies [24, 34, 41, 57], by demonstrating its role in promoting effective strategies functional in the maintenance of emotional well-being.

In other words, this study provided useful pilot data for psychological research, prevention and intervention for the general population or healthcare professionals during the COVID-19 pandemic, by implementing and proposing skills training based on these factors. Therefore, thanks to this study, greater effectiveness could be favored in addressing the negative effects on mental health due to COVID-19.
